# The Epstein-Barr Virus Episome Maneuvers between Nuclear Chromatin Compartments during Reactivation

**DOI:** 10.1128/JVI.01413-17

**Published:** 2018-01-17

**Authors:** Stephanie A. Moquin, Sean Thomas, Sean Whalen, Alix Warburton, Samantha G. Fernandez, Alison A. McBride, Katherine S. Pollard, JJ L. Miranda

**Affiliations:** a Department of Cellular and Molecular Pharmacology, University of California, San Francisco, California, USA; b Gladstone Institute of Virology and Immunology, San Francisco, California, USA; c Gladstone Institutes, San Francisco, California, USA; d Division of Biostatistics, Institute for Human Genetics, University of California, San Francisco, California, USA; e Institute for Computational Health Sciences, University of California, San Francisco, California, USA; f Laboratory of Viral Diseases, National Institute of Allergy and Infectious Diseases, National Institutes of Health, Bethesda, Maryland, USA; University of Southern California

**Keywords:** Epstein-Barr virus, transcription, chromatin, nuclear organization, latency, reactivation, Hi-C

## Abstract

The human genome is structurally organized in three-dimensional space to facilitate functional partitioning of transcription. We learned that the latent episome of the human Epstein-Barr virus (EBV) preferentially associates with gene-poor chromosomes and avoids gene-rich chromosomes. Kaposi's sarcoma-associated herpesvirus behaves similarly, but human papillomavirus does not. Contacts on the EBV side localize to OriP, the latent origin of replication. This genetic element and the EBNA1 protein that binds there are sufficient to reconstitute chromosome association preferences of the entire episome. Contacts on the human side localize to gene-poor and AT-rich regions of chromatin distant from transcription start sites. Upon reactivation from latency, however, the episome moves away from repressive heterochromatin and toward active euchromatin. Our work adds three-dimensional relocalization to the molecular events that occur during reactivation. Involvement of myriad interchromosomal associations also suggests a role for this type of long-range association in gene regulation.

**IMPORTANCE** The human genome is structurally organized in three-dimensional space, and this structure functionally affects transcriptional activity. We set out to investigate whether a double-stranded DNA virus, Epstein-Barr virus (EBV), uses mechanisms similar to those of the human genome to regulate transcription. We found that the EBV genome associates with repressive compartments of the nucleus during latency and with active compartments during reactivation. This study advances our knowledge of the EBV life cycle, adding three-dimensional relocalization as a novel component to the molecular events that occur during reactivation. Furthermore, the data add to our understanding of nuclear compartments, showing that disperse interchromosomal interactions may be important for regulating transcription.

## INTRODUCTION

The chromatin of a human interphase nucleus is structurally and functionally organized at multiple scales. Chromosomes are subdivided into topologically associated domains (TADs), structural genomic units characterized by sharp boundaries that promote long-range interactions within but not between different domains (1). On average, TADs measure ∼200 kb each (2) and fold into discrete globules in three-dimensional (3D) space (3). Each TAD may be classified into one of two large groups that correlate well with traditionally defined active euchromatin and inactive heterochromatin, based on histone modifications, gene density, and polymerase occupancy (2, 4, 5). Furthermore, TADs preferentially associate with other TADs of the same type within the same chromosome, resulting in two main compartments in the nucleus, termed compartment A and compartment B, which again roughly correspond to euchromatin and heterochromatin, respectively (2, 4, 5). These two functionally distinct partitions of each chromosome are physically separated in the nucleus (6). Assembly mediated by long-range interactions also compacts individual chromosomes so that each occupies a discrete globular space known as a chromosome territory (7). These territories similarly partition into two main groups: gene-rich chromosomes found in the center of the nucleus and gene-poor chromosomes found at the periphery (8). The organization of DNA does not necessarily proceed in a hierarchal manner but nonetheless spans scales of many orders of magnitude. Clearly, genome structure and function are linked on many levels.

Interphase chromatin is highly dynamic in a manner coupled to changes in transcription. As the transcriptional state of a TAD changes, that unit rearranges intrachromosomal contacts to associate with similarly active or inactive domains (9). Reorganization is not, however, restricted to changes in local interactions. Interchromosomal associations activate genes (10–12), and actively transcribed genes from different chromosomes colocalize (13, 14). In many cases, genes move out of a chromosome territory when they are activated (15, 16). Such long-range contacts make the spaces between chromosome territories a dynamic interface. Inhibition of transcription changes intermingling patterns of chromosome pairs, suggesting that gene activity may drive these associations (10). Taken together, these studies show that interchromosomal chromatin interactions are dynamic and that nuclear repositioning is often coupled with changes in transcription.

The human Epstein-Barr virus (EBV) is a double-stranded DNA herpesvirus that is maintained as an episome in the nucleus of a host cell. The viral genome is circular and chromatinized, resembling a small human chromosome in many molecular aspects. Like most herpesviruses, EBV establishes lifelong latency and occasionally undergoes spontaneous reactivation. The virus displays several different latent transcription programs in which different combinations of ∼10 or fewer transcripts are expressed. During reactivation, transcription drastically increases, to ∼100 transcripts, as the virus produces the proteins necessary for replication of the genome and packaging of new virions (17). Latent gene expression patterns are regulated by three-dimensional intrachromosomal interactions within the viral genome (18, 19), but how interchromosomal interactions between the virus and the human genome affect viral transcription is understudied, for technical reasons.

Since the EBV genome has a structure similar to that of human chromatin and uses similar mechanisms to control transcription at the protein level, we wondered whether the virus also uses the 3D structure and functional organization of the nucleus to regulate gene expression and the genetic switch to a very transcriptionally active state. As a first step toward answering this question, we sought to understand the extent of engagement with this nuclear organization when double-stranded DNA viruses infect cells. We used *in situ* high-throughput chromosome conformation capture (Hi-C) (2) to measure interactions between the EBV genome and the human genome during latency and reactivation. We show here that during latency, the EBV genome uses a small genetic element to interact with a network of repressive heterochromatin. Upon reactivation, the viral genome engages in different associations to leave this repressive environment and surround itself with active euchromatin.

(This article was submitted to an online preprint archive [20].)

## RESULTS

### Association of the EBV episome with the host genome depends on chromosome gene density.

We measured spatial DNA-DNA colocalization by use of *in situ* Hi-C (2) to determine how the EBV episome interacts with human chromosomes and different nuclear compartments. To maximize the signal for the transcriptionally quiescent form of the latent episome, we chose to examine Daudi, KemIII, RaeI, and Raji cells, Burkitt lymphoma cell lines that display very little spontaneous lytic reactivation capable of generating newly replicated linear genomes (21). Raji and Daudi cells contain ∼60 and ∼150 copies of the EBV episome, respectively (22). Our Hi-C data sets contain ∼17 to 40 million valid paired-end contacts after quality control filtering, of which ∼4 to 9 million are interchromosomal and ∼10,000 to 230,000 are between the EBV episome and the human genome. We observed ratios of interchromosomal to chromosomal interactions indicative of high-quality experiments that detect proximity-dependent *in vivo* colocalization instead of nonspecific *in vitro* artifacts (23).

**FIG 1:**
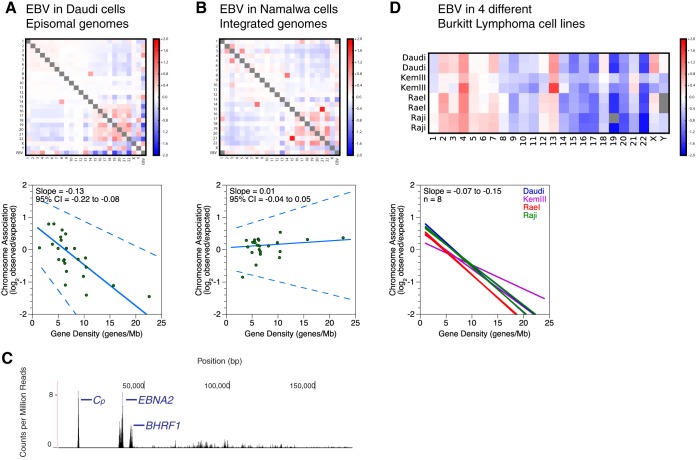
Episomal EBV genomes associate with the human genome in correlation with chromosomal gene density. (A and B) Interchromosomal contacts involving the EBV and human genomes in the Daudi and Namalwa cell lines as measured by Hi-C. Heat maps of chromosome associations between chromosomes and between each human chromosome and the EBV genome are shown. Observed counts are normalized against random expectation and shown on a log_2_ scale. Red indicates enrichment, and blue indicates depletion. Scatterplots depict virus-human chromosome associations plotted against the gene density of each chromosome. A solid line indicates the Thiel-Sen fit, and dashed lines indicate the 95% confidence interval. Results are representative of two independent biological replicates. (C) Deep sequencing of EBV transcription in the Namalwa cell line. The *x* axis denotes nucleotide position, and the *y* axis denotes the number of counts per million mapped reads. RNA signals with unambiguously assignable annotations are marked. *BHRF1*, *Cp*, and *EBNA2* (blue) are latent transcripts. Results are representative of two independent biological replicates. (D) Interchromosomal contacts between the EBV and human genomes in Burkitt lymphoma cell lines as measured by Hi-C. A heat map of chromosome associations between each human chromosome and the EBV genome in different Burkitt lymphoma cell lines is shown. Data shown are for two replicates of four different cell lines (Daudi, KemII, RaeI, and Raji). Observed counts are normalized against random expectation and shown on a log_2_ scale. Red indicates enrichment, and blue indicates depletion. Gray boxes represent either Y chromosomes not present in the female RaeI cell line or scores with absolute values of >2. In the graph at bottom, solid lines indicate the Thiel-Sen fits of virus-human chromosome associations plotted against the gene density of each chromosome. Each line represents one of two independent biological replicates for four different cell lines.

We first examined interchromosomal interactions within the human genome at chromosome-level resolution by measuring observed interactions between different human chromosomes relative to the random expectation. This metric normalizes for both random associations and chromosome length. We calculated robust and high-confidence ratios by obtaining a sequencing depth similar to that of the original Hi-C protocol that first measured chromosome-level interchromosomal interactions (5). The partitioning of gene-rich and gene-poor chromosomes into separate regions of nuclear space observed by Hi-C (5) further validates the original observations detected by fluorescence *in situ* hybridization (8). We ourselves detected small gene-rich chromosomes, such as chromosomes 16, 17, 19, and 22, preferentially associating with each other, depicted by a cluster of enriched (red) nodes in the lower right portion of the heat map in Fig. 1A. We also detected a slightly weaker preferential association between large gene-poor chromosomes, such as chromosomes 2 to 5, depicted by a second cluster of enriched (red) nodes in the upper left part of the heat map. The two groups of chromosomes tend to avoid one another, as indicated by the clusters of depleted (blue) nodes in the upper right and lower left portions of the heat map. We also noted a few strong interactions that defy this pattern. For example, the artificially high interaction frequency between chromosomes 8 and 14 is due to the chromosomal translocation commonly found in Burkitt lymphoma (24) (Fig. 1A and B; see Fig. 6A).

Next, we examined interactions of the EBV episome with human chromosomes. Based on observed/expected chromosome association measurements, we discovered that the EBV episome avoids interaction with small gene-rich chromosomes and preferentially interacts with large gene-poor chromosomes (Fig. 1A). This trend was conserved in the four cell lines we examined (Fig. 1D). Although the exact order of preference varies slightly between cell lines, the strongest ratios were observed with the EBV episome avoiding chromosomes 16, 17, 19, 20, and 22 while interacting with chromosomes 4 and 13. The observed trend does not correlate with size, based on ratios calculated for chromosomes that defy the trend of gene density increasing as size decreases. The small but gene-poor chromosome 18 is not strongly avoided by the EBV episome, and the large but gene-rich chromosome 1 does not strongly interact with the EBV episome. EBV latency type, which varies between type I for RaeI and Daudi cells and type III for KemIII and Raji cells (21), also has no effect. To illustrate the suspected trend with statistical rigor, we plotted the observed/expected chromosome association preferences for EBV against known gene density values (25) for each chromosome and calculated median slopes and 95% confidence intervals (CI) by using Thiel-Sen linear regression (26). We found a negative slope for all Burkitt lymphoma cell lines (Fig. 1D). The 95% CI falls completely in the negative range for all replicates (Fig. 1A), demonstrating that the propensity of EBV to interact with a chromosome is strongly negatively correlated with the gene density of that chromosome.

### Preferential EBV chromosome associations require episomal genomes.

To test whether viral chromosome preference is dependent on genome sequence or biophysical mobility, we performed *in situ* Hi-C on a cell line with integrated EBV. Namalwa cells contain an EBV genome with a sequence similar to others we examined, but it is integrated into chromosome 1. Gene expression predominantly consists of latent transcripts as measured by our transcriptome sequencing (RNA-seq) experiments (Fig. 1C), somewhat similar to that for the other Burkitt lymphoma lines studied. We found that integrated EBV does not show the same chromosome association preferences as episomal EBV and instead shows no correlation with gene density (Fig. 1B). The regression slope is close to 0, with the 95% CIs ambiguously spanning both negative and positive values, demonstrating that the viral genome must be episomal and less restricted to move around the nucleus in order to associate with chromosomes based on gene density. Furthermore, we note that the chromosome association preferences of integrated EBV in the Namalwa cell line are similar to chromosome 1, suggesting that the local chromatin environment at the integration site cannot be overcome by the viral sequence.

### Chromosome association preferences are conserved among some, but not all, episomal viruses.

**FIG 2:**
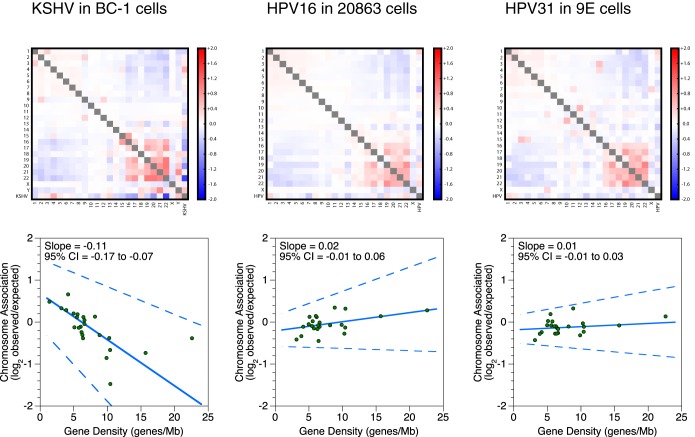
KSHV but not HPV genomes associate with the human genome in correlation with chromosomal gene density. Interchromosomal contacts involving the KSHV, HPV, and human genomes in the BC-1, 20863, and 9E cell lines were measured by Hi-C. Heat maps of chromosome associations between chromosomes and between each human chromosome and the KSHV or HPV genome are shown. Observed counts are normalized against random expectation and shown on a log_2_ scale. Red indicates enrichment, and blue indicates depletion. Scatterplots depict virus-human chromosome associations plotted against the gene density of each chromosome. A solid line indicates the Thiel-Sen fit, and dashed lines indicate the 95% confidence interval. Results are representative of two independent biological replicates.

We also performed *in situ* Hi-C on three cell lines containing other latent double-stranded DNA viruses to determine whether chromosome association preferences are a conserved feature of episomal vectors. Two lines contained different strains of human papillomavirus (HPV), HPV16 and HPV31, which are unrelated to EBV, and one line contained Kaposi's sarcoma-associated herpesvirus (KSHV), which is closely related to EBV. We found that while KSHV did show a chromosome association preference similar to that of EBV, HPV16 and HPV31 did not (Fig. 2). For KSHV, the 95% CIs for calculated linear regression slopes fall within negative values, fitting a trend of lower chromosome association as a function of gene density. For HPV, the regression slopes are close to 0, with the 95% CIs ambiguously spanning both negative and positive values. This demonstrates that while gene density-driven chromosome association preferences are not characteristic of all episomes, closely related gammaherpesviruses, such as KSHV and EBV, may use similar mechanisms to control their nuclear localization.

### OriP and EBNA1 sufficiently reconstitute preferential EBV chromosome associations.

**FIG 3:**
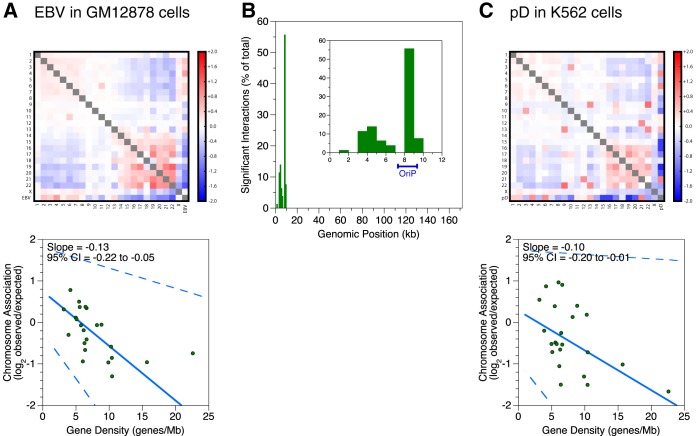
OriP is sufficient to reconstitute chromosome association preferences of full-length EBV. (A and C) Interchromosomal contacts involving EBV genomes, the pD plasmid, and human genomes in the reanalyzed GM12878 data set and the K562 cell line as measured by Hi-C. Heat maps of chromosome associations between chromosomes and between each human chromosome and the EBV genome or pD plasmid are shown. Observed counts are normalized against random expectation and shown on a log_2_ scale. Red indicates enrichment, and blue indicates depletion. Scatterplots depict virus-human chromosome associations plotted against the gene density of each chromosome. A solid line indicates the Thiel-Sen fit, and dashed lines indicate the 95% confidence interval. Results are representative of two independent biological replicates. (B) Localization within the EBV genome of significant interchromosomal contacts involving human chromosomes in the reanalyzed GM12878 data set. The histogram shows unique significant interactions between human chromosomes and the EBV episome. The percentage of total interactions was plotted against position in the EBV genome, using 1-kb bins. The inset depicts a zoomed-in view of the first 10 kb of the viral genome.

To obtain a higher-resolution understanding of which regions in the viral genome contact the human genome, we analyzed a publicly available Hi-C data set of EBV-infected B cells for which the sequencing depth far exceeds that of any other experiment, to date. The Hi-C data set obtained with GM12878 cells (2) contains ∼5 billion pairwise contacts. We would have liked all of our experiments to have been performed with a sequencing depth similar to that of the GM12878 data set, but this case represents an exceptional example in the field and is not tractably reproducible for financial reasons. We therefore complemented our own low-resolution experiments with the available high-resolution data. Our reanalysis that included the EBV genome sequence showed that viral chromosome preferences in this lymphoblastoid cell line are similar to those observed in Burkitt lymphoma lines (Fig. 3A). We then measured contact frequencies between the EBV episome and human chromosomes by using pyg (https://github.com/shwhalen/pyg), a python implementation of the Genome Organization Through Hi-C (GOTHiC) algorithm (27). We chose this simple binomial probabilistic model because we conservatively wanted to avoid potential overfitting using more complex algorithms as we undertook the novel challenge of identifying interactions between viral genomes and host chromosomes. Our analysis identified 79 significant interactions, all of which involved the first 10 kb of the EBV genome. Of these, 44 or 56% involved the 8–9 kb bin (Fig. 3B), which falls into a viral *cis*-regulatory element called OriP, genetically defined as bp 7315 to 9312.

To illuminate molecular properties of these intergenome loops, we applied the TargetFinder algorithm (28), which was previously used to determine features that predict long-range intrachromosomal interactions. We extended TargetFinder to model interchromosomal loops by requiring that one region from each interacting pair of segments be in the human genome, while the other is in the EBV genome. Features used to predict interchromosomal interactions accordingly included genomic marks on the human or EBV segment. Perhaps surprisingly, chromatin immunoprecipitation deep sequencing (ChIP-seq) signals of known chromosome organizers, such as CTCF and cohesin, on the viral genome did not significantly contribute to accurate modeling of interchromosomal loops. Among the minimal set of ∼20 to 30 features sufficient to predict interactions, binding of the viral protein EBNA1 to the EBV genome scored highly. Identification of this feature is consistent with the fact that EBNA1 binds OriP to perform two functions: replication of circular episomes during S phase and tethering of the viral genome to human chromosomes during metaphase (29). However, it is unknown whether EBNA1 mediates interactions between the viral episome and the human genome during interphase.

Considering that the majority of the significant interactions involved OriP and that EBNA1 was a top predictor of significant human-virus contacts, we hypothesized that this assembly may be involved in interphase localization of the virus. To determine if OriP DNA and the EBNA1 protein are sufficient to reconstitute the chromosome association preferences of the entire virus, we performed *in situ* Hi-C on stably transfected K562 cells containing pEBNA-DEST (pD), a plasmid that contains the sequences for these two components. We found that pD showed preferences similar to those of the entire virus (Fig. 3C). Comparing the slopes of fits for pD in K562 cells and the EBV episome in Daudi cells, we measured only statistically insignificant changes ranging from −0.03 to 0.05 (all *P* values are >0.3). The lack of a difference demonstrates that OriP and EBNA1 are sufficient to reconstitute preferential interactions with human chromosomes as seen with the full-length virus.

**FIG 4:**
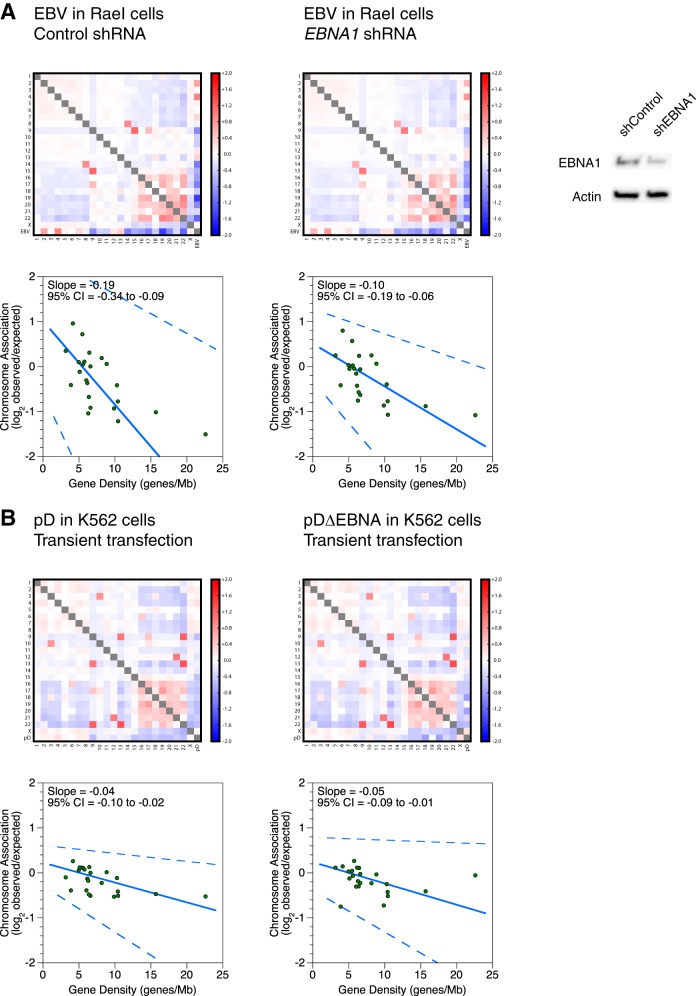
Tests of if EBNA1 is necessary to reconstitute chromosome association preferences of full-length EBV. (A and B) Interchromosomal contacts involving the pD plasmid, the pDΔEBNA plasmid, EBV genomes, and human genomes in the RaeI and K562 cell lines as measured by Hi-C. Heat maps of chromosome associations between chromosomes and between each human chromosome and the EBV genome, pD plasmid, or pDΔEBNA plasmid are shown. Observed counts are normalized against random expectation and shown on a log_2_ scale. Red indicates enrichment, and blue indicates depletion. Scatterplots depict virus-human chromosome associations plotted against the gene density of each chromosome. A solid line indicates the Thiel-Sen fit, and dashed lines indicate the 95% confidence interval. In the heat maps, a gray box off the diagonal represents a score with an absolute value of >2. (A) Lentivirus-mediated shRNA depletion of the EBV EBNA1 protein in the RaeI cell line. Western blots depict EBNA1 and β-actin expression levels in whole-cell lysates after control or EBNA1 knockdown. (B) Deletion of the EBV EBNA1 gene in the pDΔEBNA plasmid in the K562 cell line. pD and pDΔEBNA were transiently transfected prior to measurement of interchromosomal contacts by Hi-C.

We could not, however, obtain evidence that EBNA1 mediates chromosome preferences. We used short hairpin RNA (shRNA) to knock down EBNA1 in RaeI cells, which contain the full-length virus. We did not see a large change in chromosome preferences upon ∼60% knockdown (Fig. 4A). The slope of the fit changed by a statistically significant but small amount (0.02 ± 0.01 [*P* < 0.01]). We also genetically deleted *EBNA1* from pD to generate pDΔEBNA and transiently transfected both vectors into K562 cells. Transient transfections were necessary because *EBNA1* deletion precludes establishment of a stably maintained episome. The experiment is technically more variable and requires greater sequencing depth. The 95% CI of the slope fit for transiently transfected pD falls in the negative range, but the value is smaller and different from that for both replicates of full-length virus in Daudi cells (*P* < 0.01) and one of two replicates of stably transfected pD in K562 cells (*P* < 0.01 and *P* > 0.3). Nonetheless, we tested pDΔEBNA and did not see a difference in chromosome preferences between pD and pDΔEBNA: the slope changed by 0.01 ± 0.01 (*P* > 0.3) (Fig. 4B). In the context of incomplete knockdown and a synthetic transient-transfection assay, our preliminary efforts were unable to implicate EBNA1 binding to OriP in mediating chromosome association preferences.

### EBV interacts with gene-poor and AT-rich human chromatin distant from TSSs.

**FIG 5:**
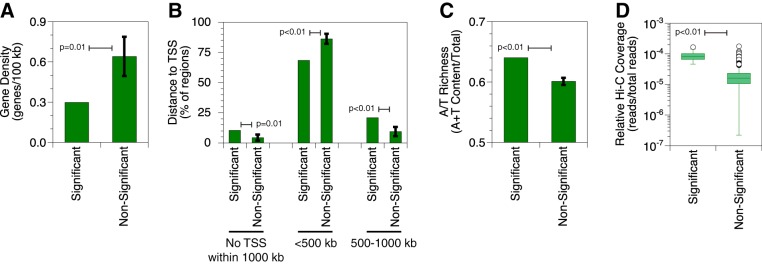
EBV episomes contact gene-poor and AT-rich human chromatin distant from transcription start sites. (A) Gene density of human genome regions that interact with the EBV episome in the reanalyzed GM12878 data set. The single set of significant interacting regions was compared to randomly chosen and equally large subsets of nonsignificant interacting regions. The error bar represents the standard deviation for 100 replicates, resulting in an empirical *P* value. (B) Distances to nearest TSSs from human genome regions that interact with the EBV episome in the reanalyzed GM12878 data set. The single set of significant interacting regions was compared to randomly chosen and equally large subsets of nonsignificant interacting regions. Error bars represent the standard deviations for 100 replicates, resulting in empirical *P* values. (C) AT content of human genome regions that interact with the EBV episome in the reanalyzed GM12878 data set. The single set of significant interacting regions was compared to randomly chosen and equally large subsets of nonsignificant interacting regions. The error bar represents the standard deviation for 200 replicates, resulting in an empirical *P* value. (D) Hi-C coverage of human genome regions that interact with the EBV episome in the reanalyzed GM12878 data set. Box-and-whisker plots are shown for relative Hi-C coverage of human genome regions that interact significantly with the EBV genome compared to the background. All significant interacting regions were compared to all nonsignificant interacting regions. Each box depicts 50% of the data, with a line indicating the median value. Whiskers extend 150% of the interquartile distance from the upper and lower quartiles, with outliers shown as circles.

To characterize the human side of chromatin interactions with latent EBV, we studied the genetic landscape of enriched sites. We again used the significant contacts from the GM12878 data set filtered through pyg. The 79 identified interactions localized to 57 unique 100-kb bins of human chromatin. We used the Genomic Regions Enrichment of Annotations Tool (GREAT) (30) to measure gene density and distances to transcription start sites (TSSs). We compared the 57 significant regions to 100 sets of 57 randomly generated nonsignificant regions. Regions of the human genome that interact with the virus have ∼50% lower gene density (empirical *P* = 0.01) (Fig. 5A). Regions that interact with EBV are 140% more likely to not have a human TSS within 1 Mb (empirical *P* = 0.01) (Fig. 5B). When present, TSSs appear ∼20% less frequently within 500 kb (empirical *P* < 0.01) and ∼120% more frequently beyond 500 kb (empirical *P* < 0.01). We performed a similar analysis to measure AT content and learned that regions interacting with EBV have an ∼10% higher AT content (Fig. 5C). This observation is consistent with previous trends correlating higher AT content with lower gene density (31, 32). We also compared significant and nonsignificant regions by using direct measures of transcriptional activity, such as RNA-seq and RNA polymerase II ChIP (data not shown). We did not detect any differences, although this may have been a result of using a 100-kb bin size, which may not have provided sufficient resolution to detect the signal. Taking these data together, we identified three properties that help to characterize the chromosomal regions with which EBV interacts as heterochromatin: being gene poor, located far from TSSs, and AT-rich.

We also used TargetFinder to ask which features on the human side were most predictive of virus-human interactions. We found that relative Hi-C coverage, when included, is the most predictive feature. In other words, regions of the human genome that interact with the viral episome have much higher Hi-C interaction frequencies than nonsignificant regions (*P* < 0.01) (Fig. 5D). This result, however, does not imply some sort of nonspecific technical artifact in our detection algorithm. We emphasize that peak calling by the GOTHiC algorithm normalizes for sequencing depth. Although the highest-coverage portions of the human genome colocalize with EBV, these regions still do so at a frequency higher than the random expectation. Moreover, regions of the EBV episome that interact with the human genome do not have higher Hi-C coverage than nonsignificant regions (*P* > 0.15) (data not shown). Together, our data argue that EBV preferentially associates with interactive, gene-poor, and AT-rich regions of human chromatin far from TSSs.

### The EBV episome switches associations from human heterochromatin to euchromatin during reactivation.

**FIG 6:**
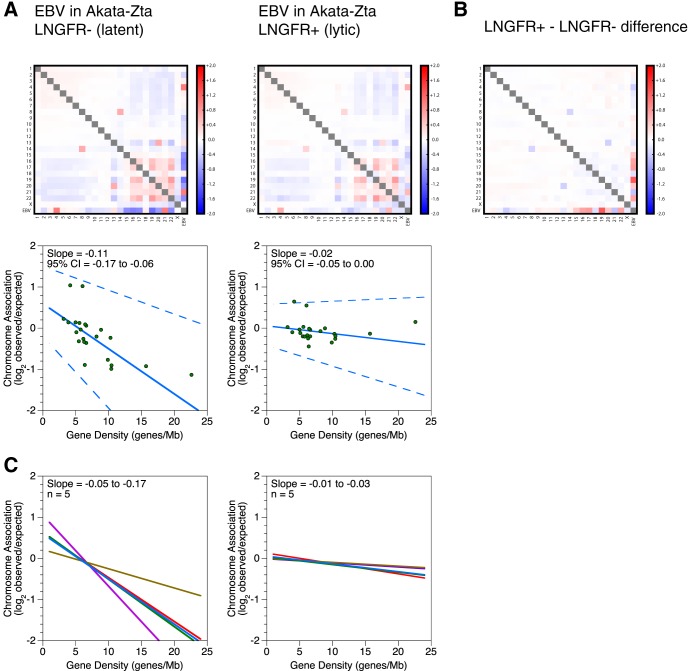
Chromosome association preferences of EBV episomes restructure during reactivation. (A) Interchromosomal contacts involving the EBV and human genomes in the Akata-Zta cell line as measured by Hi-C. LNGFR^−^ and LNGFR^+^ cells contain latent and lytic episomes, respectively. Heat maps of chromosome associations between chromosomes and between each human chromosome and the EBV genome during latency and reactivation are shown. Observed counts are normalized against random expectation and shown on a log_2_ scale. Red indicates enrichment, and blue indicates depletion. Scatterplots depict virus-human chromosome association plotted against the gene density of each chromosome. A solid line indicates the Thiel-Sen fit, and dashed lines indicate the 95% confidence interval. Results are representative of five independent and paired biological replicates. (B) Changes in interchromosomal contacts involving the EBV and human genomes in the Akata-Zta cell line upon reactivation, as measured by Hi-C. The subtraction heat map depicts differences between the latent and lytic chromosome associations shown in panel A. Chromosome association values for latency were subtracted from the values for reactivation. Results are representative of five independent and paired biological replicates. (C) Interchromosomal contacts between the EBV and human genomes in the Akata-Zta cell line as measured by Hi-C. LNGFR^−^ and LNGFR^+^ cells contain latent and lytic episomes, respectively. Solid lines indicate the Thiel-Sen fits of virus-human chromosome associations plotted against the gene density of each chromosome. Each line represents one of five independent biological replicates. Paired comparisons are matched by color.

A single snapshot of nuclear organization is often incomplete, so we measured whether interactions between the viral and human genomes remodel based on changes in transcription. To do so, we compared *in situ* Hi-C results for cells containing the latent viral episome, which expresses ∼1 to 10 transcripts, and cells containing the lytic viral episome, which expresses ∼100 transcripts (17). We used the Akata-Zta cell line, which contains the EBV genome and an additional plasmid with a doxycycline-inducible promoter that produces the viral protein BZLF1, which induces lytic gene expression, as well as nonfunctional LNGFR, which facilitates purification of reactivated cells (33). We pretreated Akata-Zta cells with acyclovir to block viral replication and to ensure that we were examining interactions of episomes and not newly replicated linear genomes. DNA deep sequencing of acyclovir- and doxycycline-treated LNGFR^−^ cells, which represent the latent population, estimated a viral genome copy number of ∼20. Acyclovir indeed functioned as intended: the total EBV DNA content increased only 2.1- ± 0.4-fold, with much of the increase coming from abortive replication attempts near the lytic origins (34). Comparison of EBV episomes in latent and lytic cells revealed that the chromosome association preferences were lost during reactivation (Fig. 6A and B). For lytic cells, linear regression slopes are close to 0, ranging from −0.01 to −0.03, instead of strongly negative, ranging from −0.05 to −0.17, as for latent cells. This loss of preferential association based on gene density was reproduced in five independent reactivation experiments, yielding large and statistically significant slope changes of 0.08 to 0.15 (all *P* values are <0.01) (Fig. 6C).

**FIG 7:**
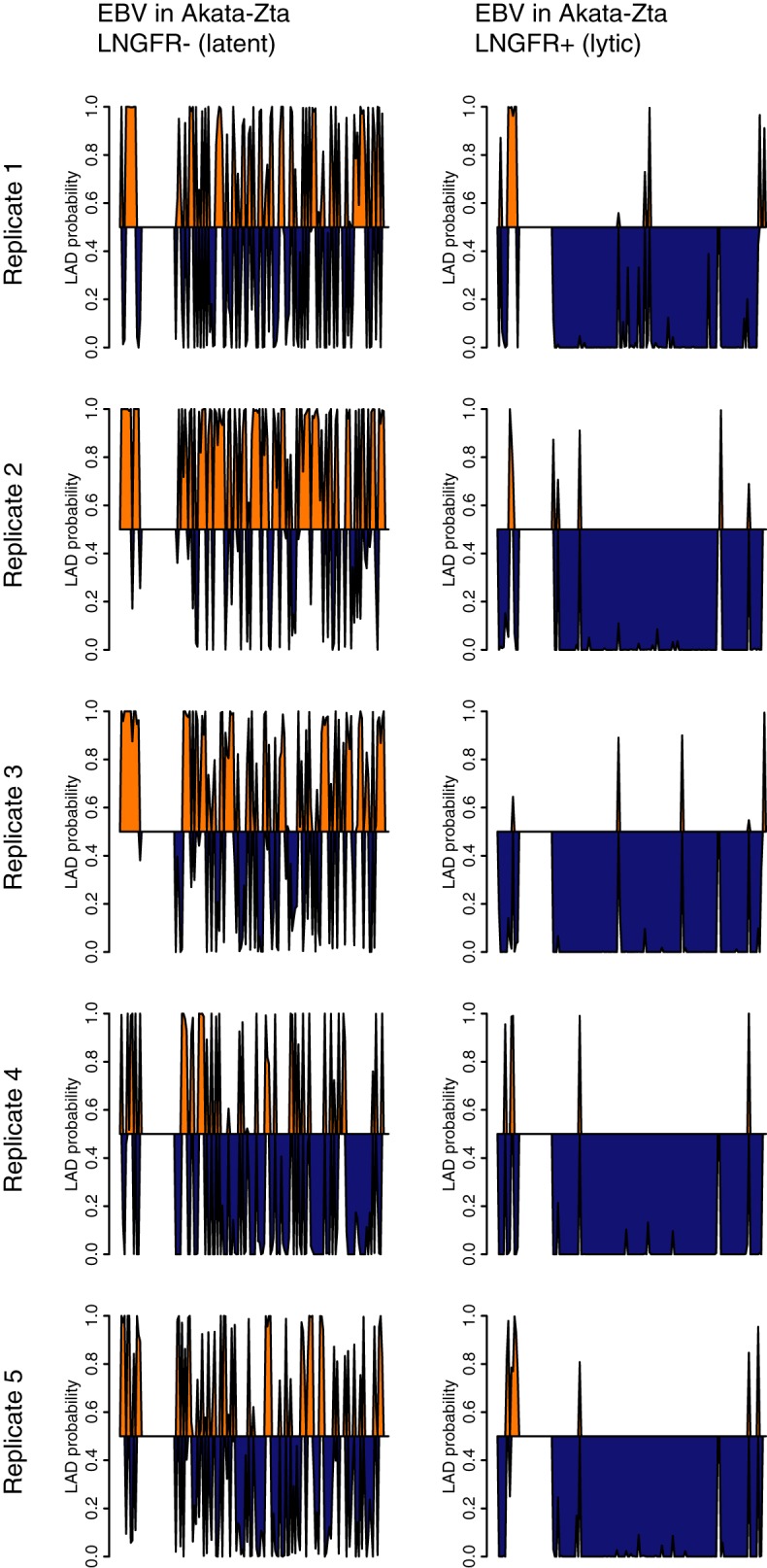
EBV episomes reorganize associations from LAD to non-LAD regions during reactivation. Predicted associations of the EBV genome with LADs during latency and reactivation in the Akata-Zta cell line are shown. LNGFR^−^ and LNGFR^+^ cells contain latent and lytic episomes, respectively. The probability of LAD association was plotted against the position in the viral genome, divided into 1-kb bins. Probabilities of >0.5 are shaded orange, and probabilities of <0.5 are shaded blue. Data shown are for five independent and paired biological replicates.

Since gene-poor chromosomes contain more heterochromatin than gene-rich chromosomes, we hypothesized that the preferential chromosome association is due to EBV localizing with specific types of host chromatin. We subsequently surmised that this interaction would change upon reactivation of the virus. For bioinformatic analysis, we used lamin-associated domains (LADs) mapped by DNA adenine methyltransferase identification (35) as markers of heterochromatin. In terms of A and B compartment classification for euchromatin and heterochromatin, respectively (2, 4, 5), LADs correspond to the B compartment (36). Different pieces of DNA in the same chromatin domain generally establish similar sets of long-range associations detectable by *in situ* Hi-C. We therefore classified bins of the human genome as either heterochromatin or euchromatin, calculated a characteristic interaction pattern for each type, and used logistic regression to determine which of these two classes the EBV genome more closely resembles. Our algorithm, LADProbs (https://github.com/geschaftsreise/LADprobs), differs from previous approaches (2, 5, 9) in that we explicitly consider interchromosomal interactions with the human genome instead of only intrachromosomal interactions. We therefore incorporate the role of the previously understudied yet prevalent associations between chromosomes and, in this case, genomes. Since heterochromatin is a transcriptionally repressive environment, we hypothesized that if viral transcription increases, associations with repressive heterochromatin and activating euchromatin will decrease and increase, respectively. We indeed found that compared to viral genomes in latent cells, episomes in reactivated cells showed a decrease in interactions with LADs and an increase in interactions with non-LAD regions (Fig. 7).

We also measured changes in the bulk amount of association between EBV episomes and human chromosomes during reactivation. For latent cells, human-virus interactions comprised 0.59% ± 0.26% of all interchromosomal paired-end contacts; for lytic cells, the percentage was 0.84% ± 0.21%. Upon reactivation, the amount of interactions did not change, yielding an insignificant increase of 69% ± 80%. EBV was therefore not merely dissociating from LADs but also shifting associations toward non-LAD regions. This suggests that the episome is leaving the transcriptionally repressive environment of heterochromatin and moving toward the transcriptionally permissive environment of euchromatin during reactivation.

## DISCUSSION

Advances in high-throughput chromosome conformation capture technologies have now allowed us to quantitatively measure molecular interactions between host chromosomes and episomal pathogen genomes. While intrachromosomal interactions are well studied, interactions between chromosomes have received little scrutiny on the genome-wide level because previous methods were not sensitive enough for thorough analysis. The original Hi-C protocol involved proximity ligation with isolated protein-DNA complexes in dilute solution outside the context of a cell (5), but the majority of interchromosomal associations detected resulted from spurious events instead of proximity-dependent intracomplex ligation (23). Recent improvements to the original Hi-C method structurally favor intracomplex ligation and yield lower rates of spurious events either through physical tethering to a surface, known as tethered Hi-C (4), or by performing the reaction within an intact nucleus, known as *in situ* Hi-C (2, 37). In this study, we used the improved signal-to-noise ratio of *in situ* Hi-C to examine the interactions between host and pathogen genomes during viral infection. We also leveraged the copy number and high-density transcriptional switching of the EBV episome to detect behavior not readily seen with autosomal loci. Our experiments reveal insight into the three-dimensional chromatin context of viral gene regulation as well as general principles about the interplay of transcription and nuclear organization.

Very little was known about positioning of the viral genome within the nucleus or its association with host chromosomes in three-dimensional space. By examining latent episomes, one previous study used three-dimensional fluorescence *in situ* hybridization after hypotonic chromosome condensation to detect possible colocalization between EBV episomes and human lamin B1 (38). That observation is consistent with our own molecular data. The same work showed EBV predominantly colocalizing with activating histone modifications and, to a lesser extent, repressive modifications. While we did not examine localization with histone modifications directly, our Hi-C data detected interactions with repressive heterochromatin during latency, an apparent contradiction with the microscopy results. Our molecular Hi-C data have a higher resolution and also examined localization with better preservation of nuclear organization by avoiding possible artifacts resulting from the hypotonic treatment used to visualize chromatin for microscopy. Another study used live-cell imaging to track nuclear positioning of lytic viral genomes during and after DNA replication but not during latency (39). Our work specifically measured associations between EBV episomes and host chromosomes for comparison during latency and reactivation.

We now know that even episomal viruses “integrate” into the network of human gene regulation. Here we showed that although EBV does not covalently integrate into the human genome, the virus noncovalently intermingles with the compartmentalized three-dimensional structure of the folded human genome. We determined that these interactions are nonrandom: the latent EBV episome preferentially interacts with gene-poor chromosomes and avoids gene-rich chromosomes. At higher resolution, the EBV episome associates with gene-poor and AT-rich regions of human chromatin distant from TSSs. The chromosome preferences can be reconstituted by OriP and EBNA1 alone. Interestingly, EBNA1 binds to regions of human chromatin with a high AT content (40). Our initial experiments, however, were unable to demonstrate that removal of EBNA1 changes chromosome preferences.

The preferential association with human chromosomes during latency is not limited to EBV. We observed similar association patterns with KSHV. The strategy is not universally used by all episomal viruses, however, as we did not detect preferential interactions between HPV and human chromatin. The cause of this distinction, perhaps rooted in different selective pressures, remains to be elucidated.

We add another layer of understanding to the sequence of molecular events coupled to viral reactivation. Much of what was previously known about the transition of EBV transcription from a latent to a lytic state involves binding of viral and host proteins to the episomal genome (19, 41). Here we show that the virus changes nuclear environments during reactivation, switching from interactions with heterochromatin during latency to interactions with the euchromatin compartment during reactivation. As a result, in both the latent and lytic transcription states, the viral genome is surrounded by human chromatin with similar transcriptional activity.

The movement of an episome upon reactivation argues that reactivation can drive passage between chromatin compartments, with changes in only a diffuse network of interchromosomal associations without strong intrachromosomal contacts. We know that transcriptional changes correlate with transitions between compartments (9). This compartment switching, however, identified changes only in the predominantly detectable intrachromosomal contacts, because previous computational methods did not consider colocalization between chromosomes (2, 5, 9). Here we show that the EBV episome changes compartments through changes in diffuse interchromosomal associations as transcription increases during reactivation. Previous examples of functional interchromosomal interactions involve single contacts between two regions (11, 12). In contrast, the EBV episome forms a myriad of associations with the heterochromatin compartment during latency and a different set of distributed associations with euchromatin after transcriptional activation. The sum of many interchromosomal interactions may therefore contribute to gene regulation.

Future studies should involve tethering the viral episome to specific compartments, though recreating the network of interchromosomal interactions detected by Hi-C will be difficult. These experiments may elucidate the functional role of nuclear localization in EBV gene regulation: forcing connections with euchromatin may induce reactivation, while anchoring connections with heterochromatin may promote latency. The role of EBV chromatin in directing or responding to nuclear localization also requires clarification. The challenge still remains to determine whether transcriptional changes drive diffuse interchromosomal colocalization or vice versa.

## MATERIALS AND METHODS

### Cell culture and plasmids.

EBV-positive Daudi, KemIII, RaeI, and Raji cells were maintained under standard conditions (21). K562 (42), EBV- and KSHV-positive BC-1 (43), and EBV-positive Namalwa (44) cells were maintained in RPMI 1640 medium with 25 mM HEPES and 2 g/liter NaHCO_3_ supplemented with 10% (vol/vol) fetal bovine serum (Invitrogen) in 5% CO_2_ at 37°C. EBV-positive Akata-Zta cells (33) were maintained in RPMI 1640 medium with 25 mM HEPES and 2 g/liter NaHCO_3_ supplemented with 10% (vol/vol) Tet system-approved fetal bovine serum (Clontech). The HPV16-positive 20863 (45) and HPV31-positive 9E (46) keratinocyte cell lines were grown in F medium, 3:1 (vol/vol) F-12–Dulbecco's modified Eagle's medium (DMEM), 5% fetal bovine serum, 400 ng/ml hydrocortisone, 5 μg/ml insulin, 8.4 ng/ml cholera toxin, 10 ng/ml epidermal growth factor, 24 μg/ml adenine, 100 U/ml penicillin, and 100 μg/ml streptomycin in the presence of irradiated 3T3-J2 feeder cells, as described previously (47). Irradiated feeder cells were removed by Versene treatment before the cells were harvested.

K562 cells were stably transfected with the pEBNA-DEST plasmid (Thermo Fisher Scientific) by use of a Nucleofector II device (Lonza) as directed, using solution V and program T-016. One day after transfection, 200 μg/ml hygromycin B was added. pD-positive K562 cells were selected until transfected cells outgrew control cells and were subsequently maintained in 200 μg/ml hygromycin B.

The *EBNA1* promoter and the majority of the coding region were deleted from pD to generate pDΔEBNA by cutting with BsgI, filling in overhangs, and ligating blunt ends. Successful construction was verified by restriction digest mapping. For transient transfections, pD or pDΔEBNA plasmids were delivered into K562 cells by use of Fugene (Promega) at a 4:1 Fugene/DNA ratio, using 20 μg DNA per 1 million cells. At 3 days posttransfection, 5 million cells were collected for Hi-C.

### *In situ* Hi-C.

*In situ* Hi-C was performed with 5 million cells per experiment as described previously (2), with slight modifications. After end repair and washes, Dynabeads (Thermo Fisher Scientific) with bound DNA were resuspended in 10 mM Tris, 0.1 mM EDTA, pH 8.0, and transferred to new tubes. Sequencing libraries were created from bound DNA by using an Ovation Ultralow library system V2 kit (NuGEN), with one modification. After adapter ligation, because DNA was still attached to the beads, water instead of SPRI beads was added to the reaction mixture. Beads with bound DNA were purified by use of a magnet, washed, and resuspended in 10 mM Tris, 0.1 mM EDTA, pH 8.0. After library amplification, SPRI beads were added as directed to purify the amplified DNA. Quantitation and size distribution of libraries were performed using a Bioanalyzer High Sensitivity DNA kit (Agilent). Fifty-base paired-end reads were sequenced on a HiSeq instrument (Illumina).

Once sequenced, paired reads were aligned to combined human/viral reference genomes by use of the Hi-C User Pipeline (HiCUP), version 0.5.0, using default parameters (48) to generate a set of interactions. We used the human hg19 sequence merged with the EBV (accession no. NC_007605.1), KSHV (accession no. NC_009333.1), HPV16 (accession no. NC_001526.2), or HPV31 (accession no. J04353.1) sequence. The HiCUP processing steps remove PCR duplicates as well as invalid read pairs, including those that are self-ligated or map to identical or adjacent fragments. Only alignments with mapq scores of ≥30 were retained. Data sets contained ∼7 to 40 million valid paired-end Hi-C contacts after quality control filtering, of which ∼2 to 20 million were interchromosomal and ∼400 to 230,000 were between human and viral or plasmid sequences.

### Analysis of interchromosomal interactions.

Chromosome-resolution heat maps of interactions were determined from the HiCUP-filtered interchromosomal Hi-C interactions in sam format. Expected interactions were calculated using Equation 1 for each chromosome pair:

(1) $$\left[\left(\frac{\text{chrA}}{\text{all}}\times \frac{\text{chrB}}{\text{all}-\text{chrA}}\right)+(\frac{\text{chrB}}{\text{all}}\times \frac{\text{chrA}}{\text{all}-\text{chrB}})\right]\times \text{totalPairs}$$

where “chrA” represents the number of single-end interchromosomal reads containing chromosome A, “chrB” represents the number of single-end interchromosomal reads containing chromosome B, “totalPairs” represents the total number of interchromosomal paired-end reads, and “all” represents the total number of interchromosomal single-end reads, which is equal to 2 × totalPairs.

The chromosome association preference value for each combination was calculated by dividing the observed number of reads containing chrA and chrB by the expected value. Chromosome association preferences of viral genomes were plotted against gene density, measured in genes per megabase (25). Data were fit to a line by using the Thiel-Sen nonparametric linear regression median slope method (26) as implemented in the zyp R package. Comparison of slopes was performed by fitting a new slope to the difference for two samples and calculating the *P* value from the confidence intervals (49).

### ChIP-seq.

BZLF1 and EBNA1 ChIP-seq experiments were performed as previously described (34), using 3 μg of the anti-BZLF1 antibody BZ1 (sc-53904; Santa Cruz) and 3 μg of the anti-EBNA1 antibody 0211 (sc-57719; Santa Cruz).

### Analysis of virus-human contact regions.

A large Hi-C data set from GM12878 cells (2) was reanalyzed to identify the strongest interactions between the EBV episome and human chromosomes. Chromosome-resolution heat maps of interactions and chromosome association preference plots were generated from the HIC001 library. Further reanalysis included all fastq files from the primary and replicate sets (libraries HIC001 to HIC0029). Data were independently processed using HiCUP, version 0.5.9, and significant looping interactions were called using pyg (https://github.com/shwhalen/pyg), a Python implementation of the GOTHiC algorithm (27). Our specific parameters for performing the GOTHiC algorithm in *trans* mode were as follows. Paired-end reads with at least one fragment mapping to EBV were retained, but those containing alignments to mitochondrial DNA were discarded. To increase statistical power, reads were first mapped to fixed-resolution bins (100-kb bins on the human genome and 1-kb bins on the viral genome) by using pairToBed as packaged in BEDTools, version 2.26 (50). Counts from the two biological replicates were merged to increase the signal-to-noise ratio. Next, the probability of a spurious ligation was computed as a function of the relative coverage of each bin. Relative coverage was defined as the number of reads mapping to the bin divided by the total number of reads. Finally, the probability of observing a given number of reads by chance between two bins was computed using a binomial test, resulting in a *P* value for each pair of bins. Multiple-testing correction was performed using the Benjamini-Hochberg procedure, resulting in a *q* value for each pair of bins. Bin pairs with a *q* value of 0.05 or less, which corresponds to a 5% false-discovery rate, were treated as statistically significant and identified as positive interacting samples.

Features that predict colocalization between the EBV episome and human chromosomes were identified using the TargetFinder algorithm (28). Average signals for all ENCODE GM12878 ChIP-seq data sets, BZLF1 and EBNA1 ChIP-seq experiments performed by us, and AT content and GC content were computed for EBV and human bins. Separate calculations were performed with and without relative Hi-C coverage included. These features were used with interaction labels to train a gradient boosting classifier by using the scikit-learn Python package (51) with the following parameters: n_estimators = 1,000, learning_rate = 0.01, and max_depth = 2. Stratified 10-fold cross-validation was performed with scikit-learn to obtain scores for precision, recall, and F1, the harmonic mean of precision and recall.

We characterized the gene density and TSS landscape of human chromosomal regions that colocalize with the viral episome by using GREAT (http://great.stanford.edu/public/html/) (30). To measure gene density, the “basal plus extension” parameter was used, with a proximal extension of 50 kb upstream and 50 kb downstream and a distal extension of 0 kb. Since GREAT chooses the midpoint of the 100-kb human bin as a reference, these settings allowed genes to overlap and permitted measurement of the total number of TSSs in each region. To determine distances to TSSs, the “single nearest gene” parameter was set to search within 1,000 kb, allowing determination of the nearest TSS in either direction. Each of these two analyses was performed on the 57 significant bins and on 100 sets of 57 random nonsignificant bins. An empirical *P* value was measured to determine significance. We measured AT content by using nuc as packaged in BEDTools, version 2.26 (50). This analysis was performed on the 57 significant bins and on 200 sets of 57 random nonsignificant bins. Student's *t* test was applied to compare the distribution of relative coverage values in the sets of significant and nonsignificant bins.

### shRNA-mediated EBNA1 knockdown.

RaeI cells were transduced with shEBNA1 or control shRNA (52) by spinoculation. Lentivirus with 8 μg/ml Polybrene was added to cells and spun at 800 × *g* for 30 min at room temperature. The supernatant was aspirated and cells resuspended in fresh medium. At 48 to 72 h posttransduction, RaeI cells were selected with 2 μg/ml puromycin. At 7 days posttransduction, cells were collected for Western blotting and 5 million cells were collected for *in situ* Hi-C.

Western blots were performed using standard techniques. EBNA1 was detected using the anti-EBNA1 antibody 1EB12 (sc-81581; Santa Cruz) at a 1:100 to 1:200 dilution and goat anti-rabbit–horseradish peroxidase (HRP) (ab721; Abcam) at a 1:2,000 to 1:5,000 dilution. For normalization, actin was detected using an anti-β-actin antibody (ab8227; Abcam) at a 1:10,000 to 1:20,000 dilution and rabbit anti-mouse–HRP (ab6728; Abcam) at a 1:20,000 to 1:30,000 dilution. Signals were detected using SuperSignal West Pico chemiluminescence substrate (Thermo-Fisher) and a ChemiDoc MP imaging system (Bio-Rad). ImageLab (Bio-Rad), version 5.2.1, was used to measure the knockdown level.

### Viral reactivation.

Log-phase cultures of Akata-Zta cells were pretreated with 200 μM acyclovir (Sigma-Aldrich) for 1 h before reactivation of the lytic cycle with 500 ng/ml doxycycline (Sigma-Aldrich). These cells contain a doxycycline-inducible plasmid with a bidirectional promoter that produces nonfunctional LNGFR along with the immediate early protein BZLF1, which starts the lytic cycle gene expression cascade and reactivates the virus (33). After 1 day, cells were magnetically sorted using LNGFR microbeads and LS columns (Miltenyi Biotech).

EBV DNA quantitation was determined by deep sequencing of total DNA. Genomic DNA was purified by silica-based membrane affinity purification with a DNeasy blood and tissue kit (Qiagen). Libraries were constructed, and the percentage of EBV reads was measured (34). The viral genome copy number was estimated based on the observed ratio between the numbers of reads mapped to the EBV and human genome sequences and the expected value, calculated as the ratio between the viral episome length and the summed length of all human chromosomes.

### LAD state predictions.

The set of lamin interacting domains from Tig3 cells (35) was downloaded from the UCSC genome browser (53). Hi-C interactions identified by HiCUP were processed into a format readable by the HiTC package (54), using 1-Mb bins for the human genome and 1-kb bins for the viral genome. We performed further analyses of these interaction matrices in R (34). First, a full interaction matrix for all autosomes was constructed. Next, for each bin, the mean of interaction counts for LAD bins was calculated, as was the mean of interaction counts for non-LAD bins. This created two vectors of interaction means, whose lengths were the numbers of bins in the autosomal genome. These vectors contained the primary source of information linking the LAD state and interaction counts. Correlations were then performed across all autosomal bins by using the LAD mean vector and the non-LAD mean vector. A LAD bin will have a high correlation of interactions with the LAD mean counts and a low correlation of interactions with the non-LAD mean counts. A logistic regression was therefore performed on the LAD and non-LAD correlation values to estimate the probability of each genome region interacting with lamin. Next, we calculated the probability that each viral bin interacted with the lamin by applying the logistic regression model of the autosomal LAD correlations to the viral interaction data. The R function, called LADprobs, and associated files used to perform this calculation are available for download (https://github.com/geschaftsreise/LADprobs).

### Accession number(s).

Deep-sequencing data were deposited in the Gene Expression Omnibus database under accession number GSE98123.
